# Supplementary figures and images for: Translocation of Drought-Responsive Proteins from the Chloroplasts
Source: Cells. 2020 Jan 20;9(1):259. doi: 10.3390/cells9010259 (PMC7017212; doi:10.3390/cells9010259)

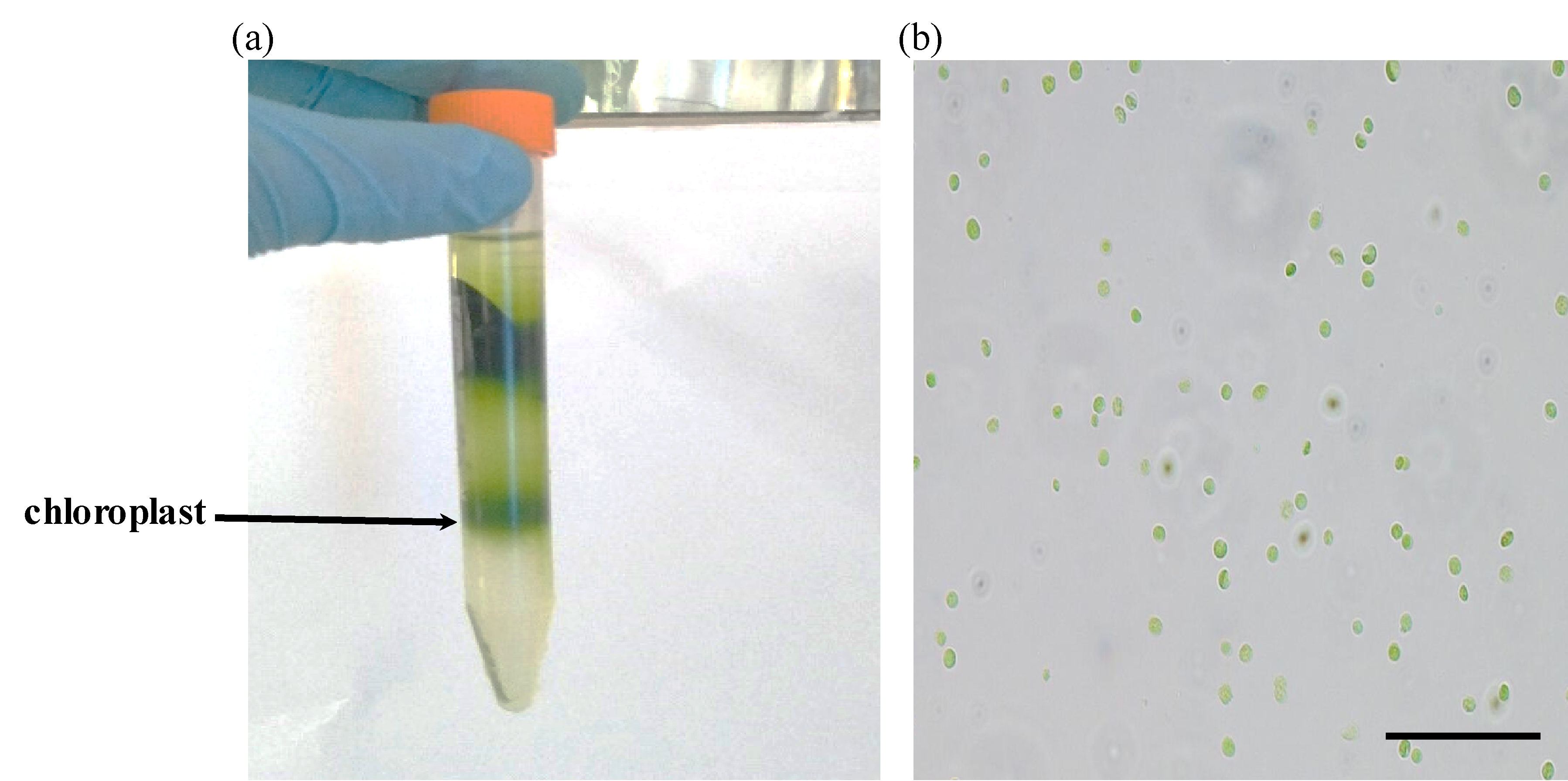

Supplement: Supplementary file 1 [file cells-09-00259-s001.zip › supplementary materials/Figure S1.tif]

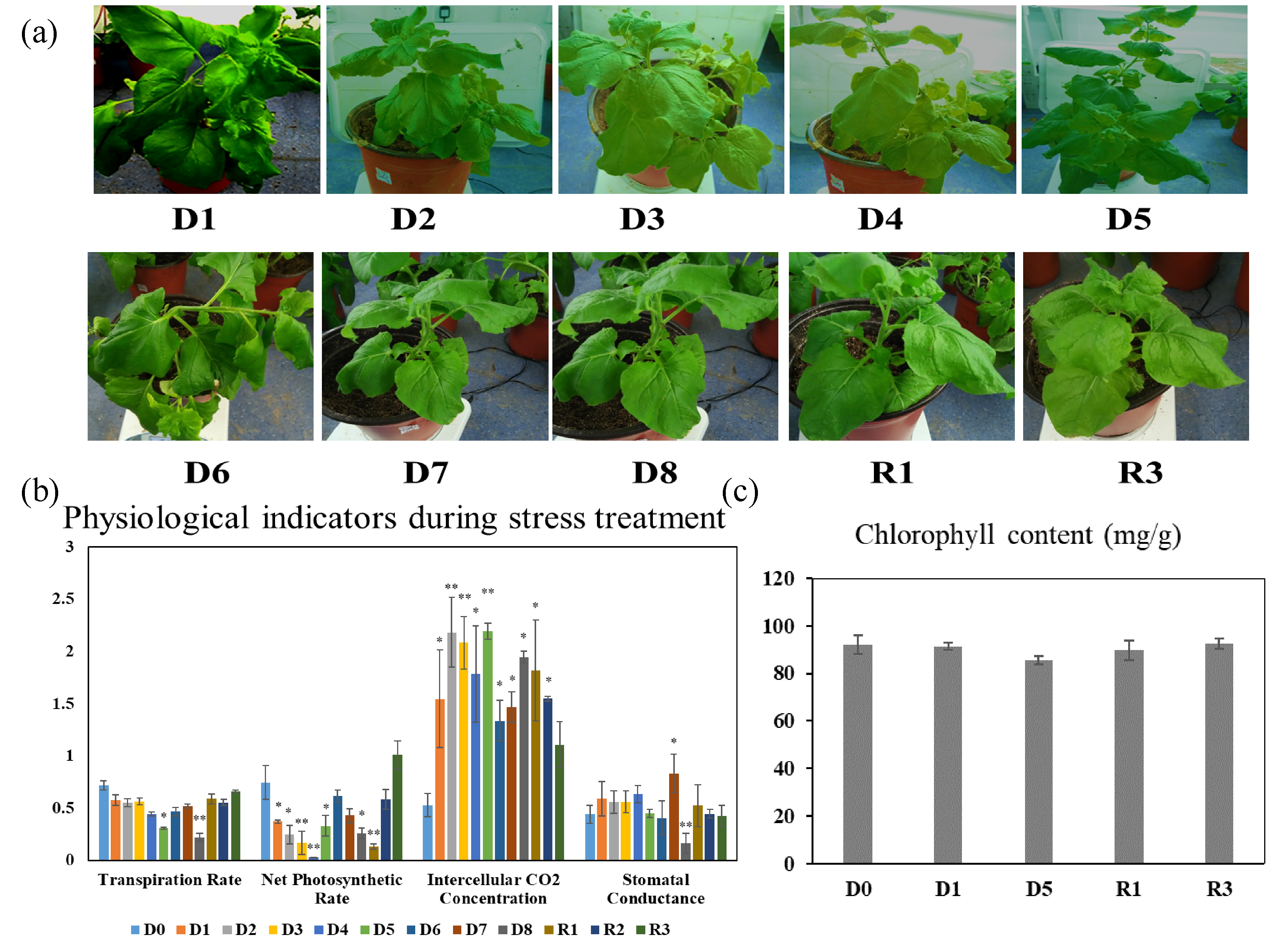

Supplement: Supplementary file 1 [file cells-09-00259-s001.zip › supplementary materials/Figure S2.tif]

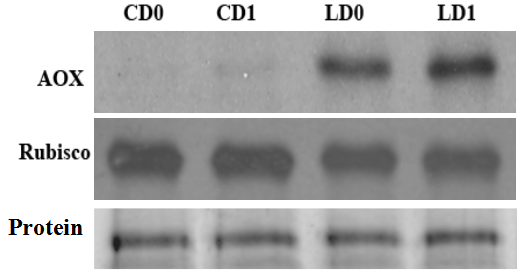

Supplement: Supplementary file 1 [file cells-09-00259-s001.zip › supplementary materials/Figure S3.tif]

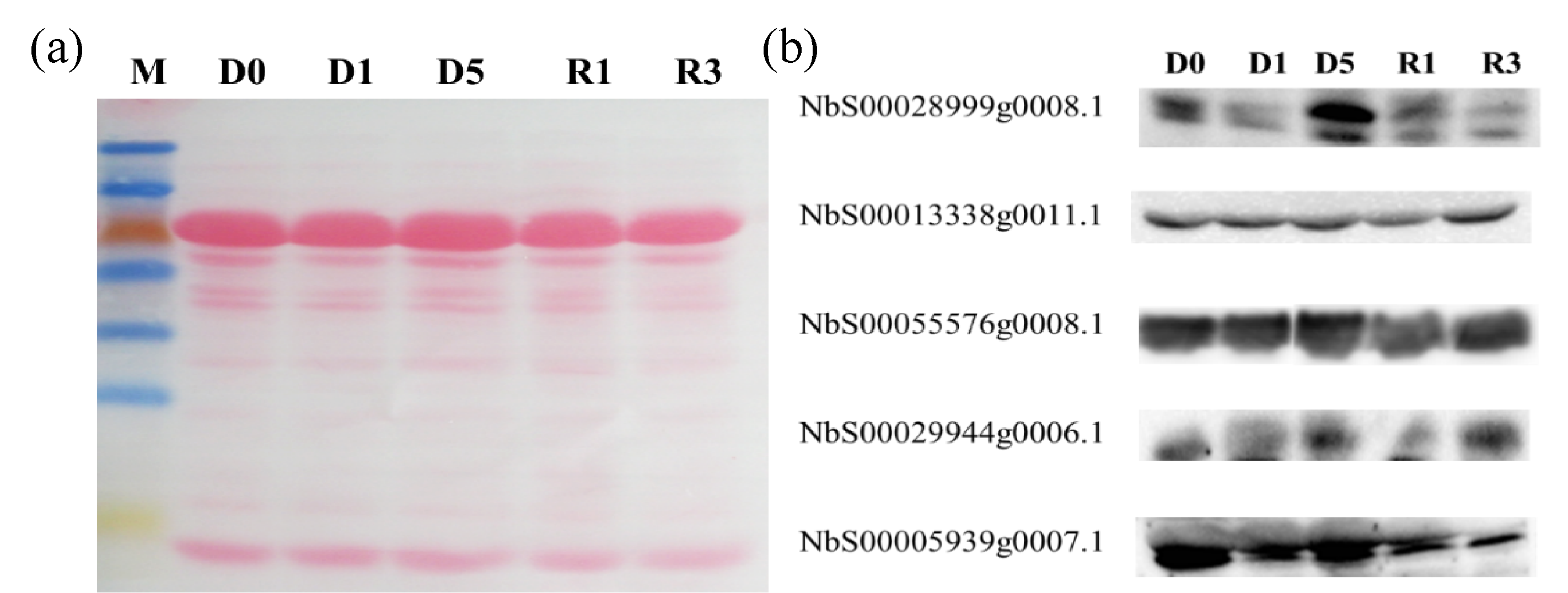

Supplement: Supplementary file 1 [file cells-09-00259-s001.zip › supplementary materials/Figure S4.tif]

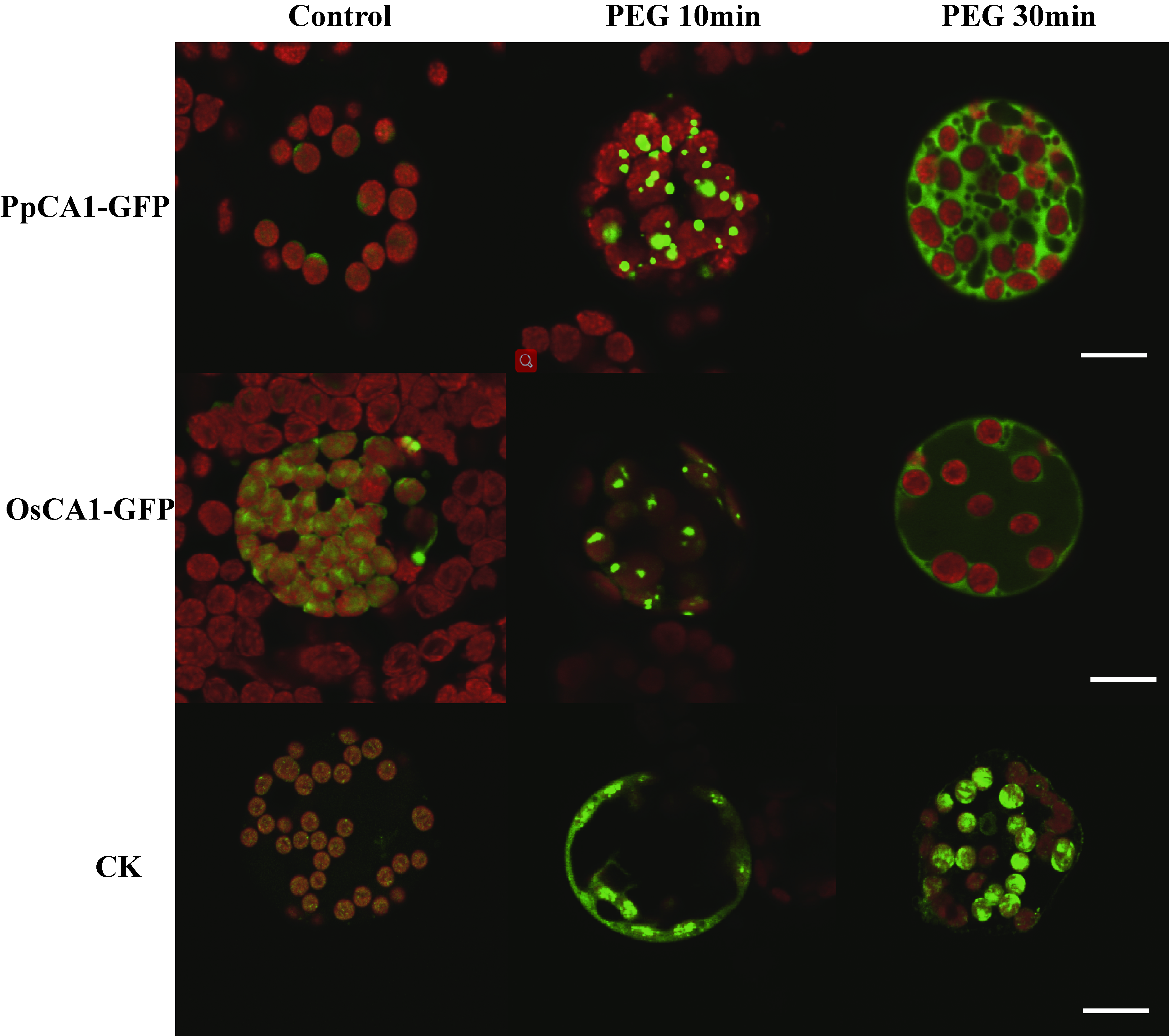

Supplement: Supplementary file 1 [file cells-09-00259-s001.zip › supplementary materials/Figure S5.tif]

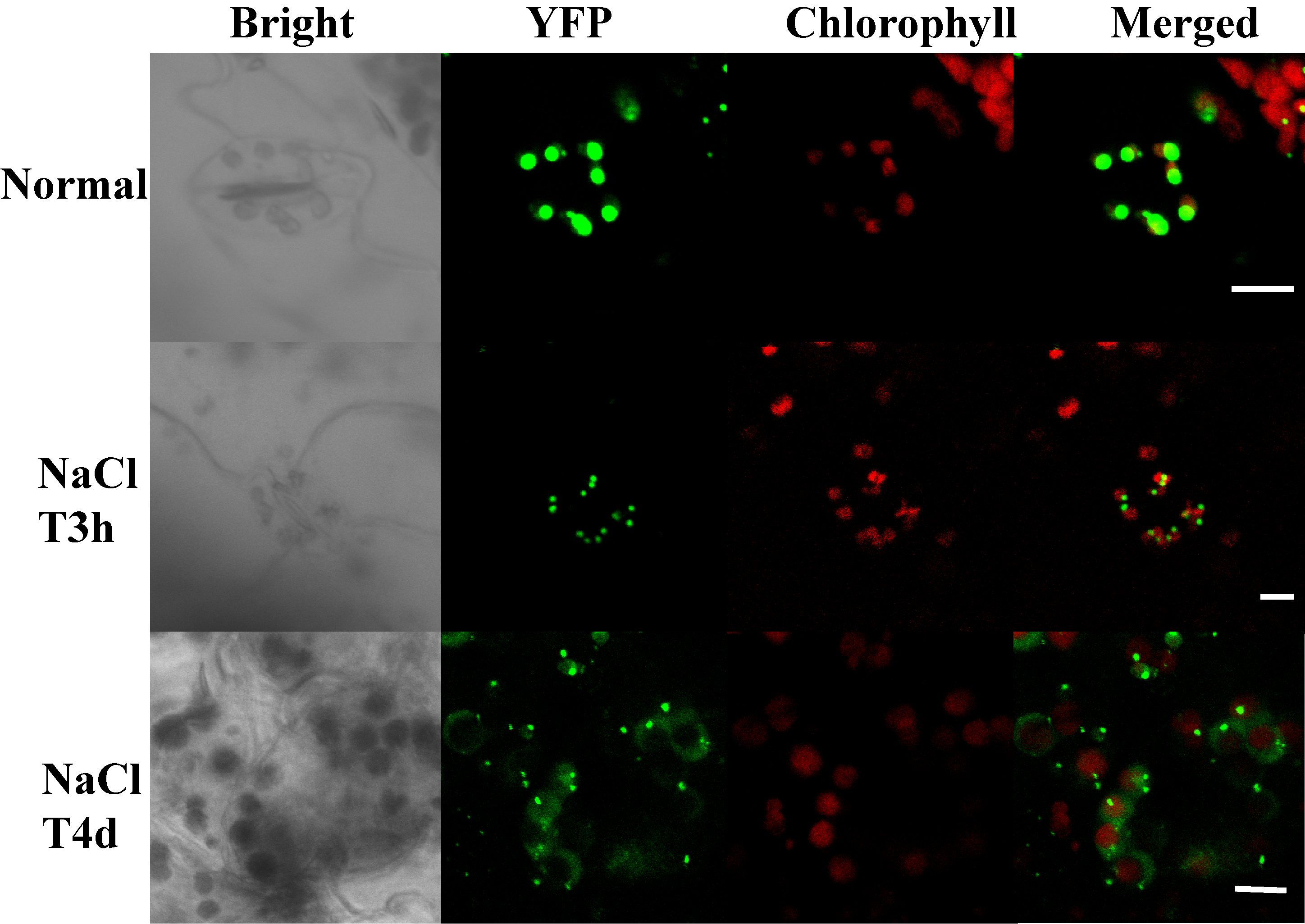

Supplement: Supplementary file 1 [file cells-09-00259-s001.zip › supplementary materials/Figure S6.tif]
